# Case Report: Two clinical cases of Wilms tumor comorbid to gingival fibromatosis in young children with constitutionally mutated *REST*

**DOI:** 10.3389/fonc.2023.1192489

**Published:** 2023-06-22

**Authors:** Anastasiya S. Salomatina, Liudmila A. Yasko, Maria A. Kurnikova, Yulia M. Mareeva, Ruslan K. Abasov, Nina V. Gegeliya, Anna M. Mitrofanova, Natalia Y. Usman, Galina A. Novichkova, Alexander E. Druy

**Affiliations:** ^1^ Dmitry Rogachev National Medical Research Center of Pediatric Hematology, Oncology and Immunology, Moscow, Russia; ^2^ Research Institute of Medical Cell Technologies, Yekaterinburg, Russia

**Keywords:** Wilm’s tumor, REST (RE-1 silencing transcription factor), pediatric oncology, gingival fibromatosis, next-generation sequence (NGS)

## Abstract

**Introduction:**

Nephroblastoma (Wilms tumor (WT)) is an embryonal tumor accounting for >90% of pediatric renal cancers. About 10% of WTs harbor pathogenic germline mutations. The *REST* gene, classified as a putative tumor suppressor, is affected in 2% of WTs. High-throughput molecular methods facilitate advanced diagnostics of cancer. In addition to this, germline mutations in *REST* are also associated with familial gingival fibromatosis (GFM). Reciprocally, none of the articles on *REST*mut WT mentions GFM as a comorbid condition. This report provides unique evidence on the WT-GFM comorbidity in *REST*mut carriers.

**Case presentation:**

Patient 1 (a 5-year-old boy with unilateral WT) is a proband, who has two healthy siblings. Patient 2 (a 4-year-old girl with bilateral WT) is a proband from *in vitro* fertilization (IVF) triplets, with a sister and brother without WT. We analyzed probands’ DNA extracted from peripheral blood leucocytes with a custom-targeted next-generation sequencing (NGS)-198 gene panel. The detected variants were checked in family members by Sanger sequencing. Patient 1 had a pathogenic germline mutation in *REST:* c.1035_1036insTA, p.(E346*), as did his mother and both brothers. There were two other WT cases in this family (proband’s maternal uncles). Patient 2 had a pathogenic germline variant in *REST*: c.2668_2671del, p.(E891Pfs*6), as well as her sister. The mutation was probably inherited from their deceased father, as he had gingival fibromatosis. Family members with *REST* mutations from both families had gingival fibromatosis. A somatic *REST* c.663C>A p.C221* mutation was identified in one patient with WT. At the moment both patients with WT are under dynamic observation without signs of the disease.

**Conclusion:**

Here, we describe two clinical cases of WT in nonrelated young children with germline-inactivating *REST* variants identified by next-generation sequencing. Both patients present with familial gingival fibromatosis, regarded as clinically useful comorbidity indicative of the tumor predisposition syndrome. The two cases illustrate Wilms tumor-gingival fibromatosis comorbidity in carriers of germline-inactivated *REST* alleles previously identified as a predisposition factor for both conditions.

## Introduction

Nephroblastoma, a.k.a. Wilms tumor (WT), is embryonal-type renal cancer developing from pluripotent progenitor cells of primordial kidneys (1).

At an estimated prevalence of 0.7–1 per 100,000 in pediatric populations, WT accounts for 5% of pediatric cancer cases. About 90% of pediatric kidney tumors are WTs, and about 80% of WTs are diagnosed in under-5-year olds. The median age at diagnosis is 3.5 years (2, 3).

Although WTs are mostly sporadic, 10% of them involve hereditary predisposition (4). Congenital genetic conditions associated with high risks of WT are listed in **Box 1**; clinical manifestations range from multiple congenital malformations to the lack of a distinguishable phenotype apart from cancer risks. WT predisposition can be suspected based on genitourinary malformations, bilateral primary lesions (encountered in up to 10% of WTs), and multifocal patterns, as well as the early onset and family history of renal tumors (4, 5).

**Box 1 Wilms tumor (WT) predisposition syndromes.**

Most significant in terms of prevalence and risks:

 WT1-associated syndromes:

 Denys–Drash syndrome (AD^*^) (OMIM: 607102): WT1 (11p13)

 Frasier syndrome (AD) (OMIM: 607102): WT1 (11p13)

 Despite a degree of clinical dissimilarity attributed to different patterns of mutagenesis within WT1 coding region, these syndromes reveal a continuum of clinical signs qualifying them as a single nosological entity.

 PAX6 and WT1 deletion syndrome (11p13 del) a.k.a. WAGR (Wilms tumor-aniridia-genital anomalies-retardation/plus obesity (WAGRO)) (OMIM 194072, 612469)

 Beckwith–Wiedemann syndrome (AD, uniparental disomy, epimutations mapped to 11p15.5, e.g., in CDKN1C) (OMIM: 616186, 604115, 600856)

 Other:

 Fanconi anemia (AR) (OMIM: 227650): BRCA2, PALB2

 Trisomy 18 (Edwards syndrome)

 TP53-dependent tumor predisposition (Li-Fraumeni syndrome) (AD) (OMIM: 191170): TP53

 GLOW syndrome (AD, SM) (OMIM: 618272): DICER1

 Perlman syndrome (AR) (OMIM: 614184): DIS3L2

 Bohring–Opitz syndrome (AD) (OMIM: 605039): ASXL1

 Bloom syndrome (AR) (OMIM: 210900): BLM

 Simpson–Golabi–Behmel syndrome (XLR) (OMIM: 180849): GPC3, GPC4

 Mulibrey (MUscle, LIver, BRain, and EYes) nanism (AR) (OMIM 605073): TRIM37

 Mosaic variegated aneuploidy syndrome (AR) (OMIM: 257300): BUB1B, TRIP13

 PIK3CA-related overgrowth spectrum (SM) (OMIM 613089, 612918, 602501): PIK3CA

 WT, REST^mut^-associated (AD) (OMIM: 616806)

 WT, CTR9^mut^-associated (AD)

 WT, TRIM28^mut^-associated (AD)

 Int. J. Cancer: 146, 1010–1017 (2020).

 NCCN Clinical Practice Guidelines in Oncology (NCCN Guidelines®) Wilms Tumor (Nephroblastoma) Version 1.2022—12 April 2022

 Human Molecular Genetics, Volume 29, Issue R2, 1 October 2020, Pages R138–R149.

 
^*^Inheritance patterns: AD, autosomal dominant; AR, autosomal recessive; XLR, X-linked recessive; SM, somatic mosaicism.

High-throughput molecular methods facilitate the discovery of nonconventional tumorigenic mutations in solid tumors, including recently described *CTR9*, *TRIM28*, and *REST* variants in WT (6, 7). ‘Restrictive element-1 silencing transcription factor’ (*REST*) encodes a zinc-finger nuclear protein. Mahamdallie et al. (2015) (6) describe 11 germline-inactivating *REST* variants in patients with WT. However, initially, pathogenic variants in *REST* were associated with familial gingival fibromatosis (GFM), as researchers mentioned in their studies (8).

Here, we report for the first time WT comorbidity to GFM in nonrelated young children with constitutionally mutated *REST*.

## Clinical case descriptions

### Patient 1

The boy, 4 years old, was born from a second pregnancy by a second full-term delivery. Early postnatal development was normal. A scheduled ultrasound examination after the appendectomy identified a mass in the lower pole of the right kidney. The patient received 4-week actinomycin D and vincristine (AV) neoadjuvant chemotherapy under the SIOP-RTSG UMBRELLA 2016 protocol, followed by surgical treatment. The tumor was identified as “nephroblastoma, regressive type, histologically intermediate risk group, local stage 1, 99.5% necrosis.” The residual vital tumor comprised small, solitary blastemal foci. Postoperatively, Pt 1 received 4 weeks of AV adjuvant chemotherapy. The patient has completed specific treatment and is under dynamic observation for 19 months without signs of the disease.

The patient has a burdened family history: one maternal uncle of the proband died from a renal tumor at the age of 3; a maternal aunt died of an unknown cause as a child; another maternal uncle and a maternal cousin–uncle underwent surgery for kidney masses at the age of 1.5–2 years.

**Figure 1:**
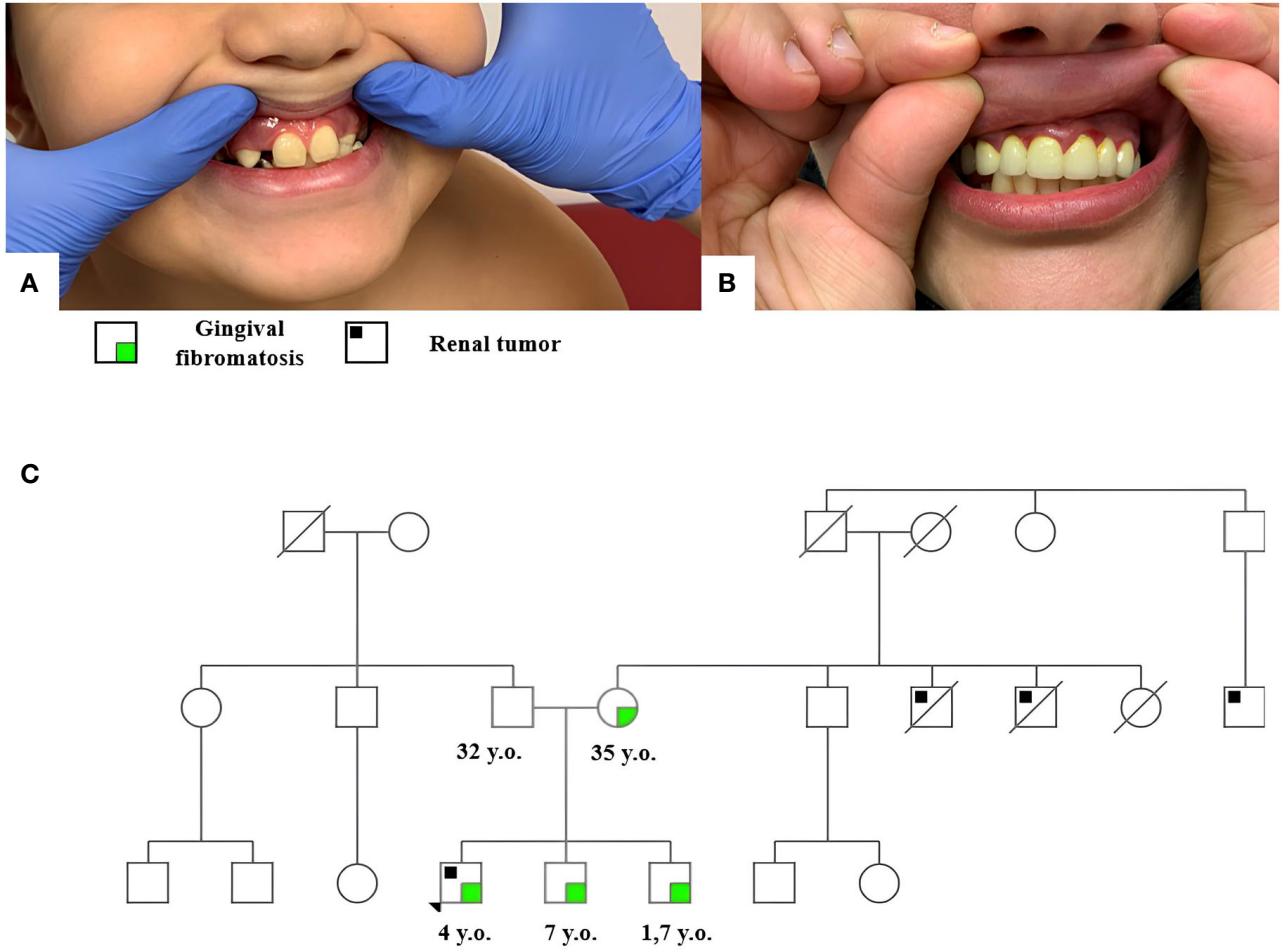
Gingival fibromatosis in patient 1 **(A)** and his mother **(B)**; the family tree of patient 1 **(C)**.

Clinical examination revealed hyperplasia of the maxillary gums typical of GFM (**Figure 1A**). Similar hyperplastic changes are observed in the mother (**Figure 1B**) and both brothers of the patient. The family tree is shown in **Figure 1C**.

The burdened family history suggested a hereditary cancer predisposition in the patient. Peripheral blood DNA was analyzed by high-throughput NGS using a tumor predisposition syndrome target panel (Roche, USA) of genes listed in **Supplementary Table S1**. The analysis revealed a *REST* (RefSeq NM_005612) c.1035_1036insTA, p.E346* heterozygous mutation qualified as pathogenic according to the American College of Medical Genetics (ACMG) guidelines. Subsequent genetic testing identified this constitutional variant in the patient’s mother and his two brothers, 7 years and 19 months old, both of whom presented without tumors but with signs of gum hyperplasia at the time of this writing.

To test for the additional tumorigenic event(s), the residual vital foci of blastemal tissue were microdissected from histological slides for DNA extraction, followed by Klenow-assisted whole-genome amplification. NGS (QIAseq, Qiagen, Germany) at an average depth of 4,197 × identified somatic *REST* c.663C>A, p.C221* mutation at a 6% allele frequency read 9,176 times. The list of genes included in the NGS panel for somatic sequencing is presented in **Supplementary Table S1**.

Thus, the tumor harbored two stop mutations in *REST*, one germline and one somatic, apparently in transconfiguration (one mutation per copy), leading to the loss of REST protein in tumor cells.

### Patient 2

The girl, 3 years old, was born from the sixth pregnancy (IVF-induced, triple) by surgical delivery on gestation week 31. The neonatal period was complicated by bacterial pneumonia, hypoxic–ischemic CNS damage, and muscular dystonia syndrome. The patient’s triplet sister is under observation for cerebral palsy; the other sibling (a boy) is healthy.

A scheduled ultrasound examination of abdominal organs revealed a mass in the left kidney. Additional examination revealed lesions in the other kidney. The patient received AV neoadjuvant chemotherapy followed by bilateral nephron-sparing surgery. Histological assessment of the left kidney specimen revealed foci of diffuse perilobar nephroblastomatosis as well as renal mass identified as “nephroblastoma, regressive type, 90% necrosis.” The residual vital tumor was presented with 90% blastemal and 10% epithelial components. The right kidney tumor was entirely blastemal without signs of response to treatment. The patient was diagnosed with “bilateral nephroblastoma, stage V.” She had a histologically intermediate-risk group of the left kidney, local stage 1, and a histologically high-risk group of the right kidney, local stage 1. The surgical margins of resection for each specimen were microscopically negative. Postoperatively, the patient received 27 weeks of adjuvant chemotherapy of actinomycin D, vincristine, and doxorubicin (AVD). The patient has completed specific treatment and has been under dynamic observation for 17 months without signs of the disease.

**Figure 2:**
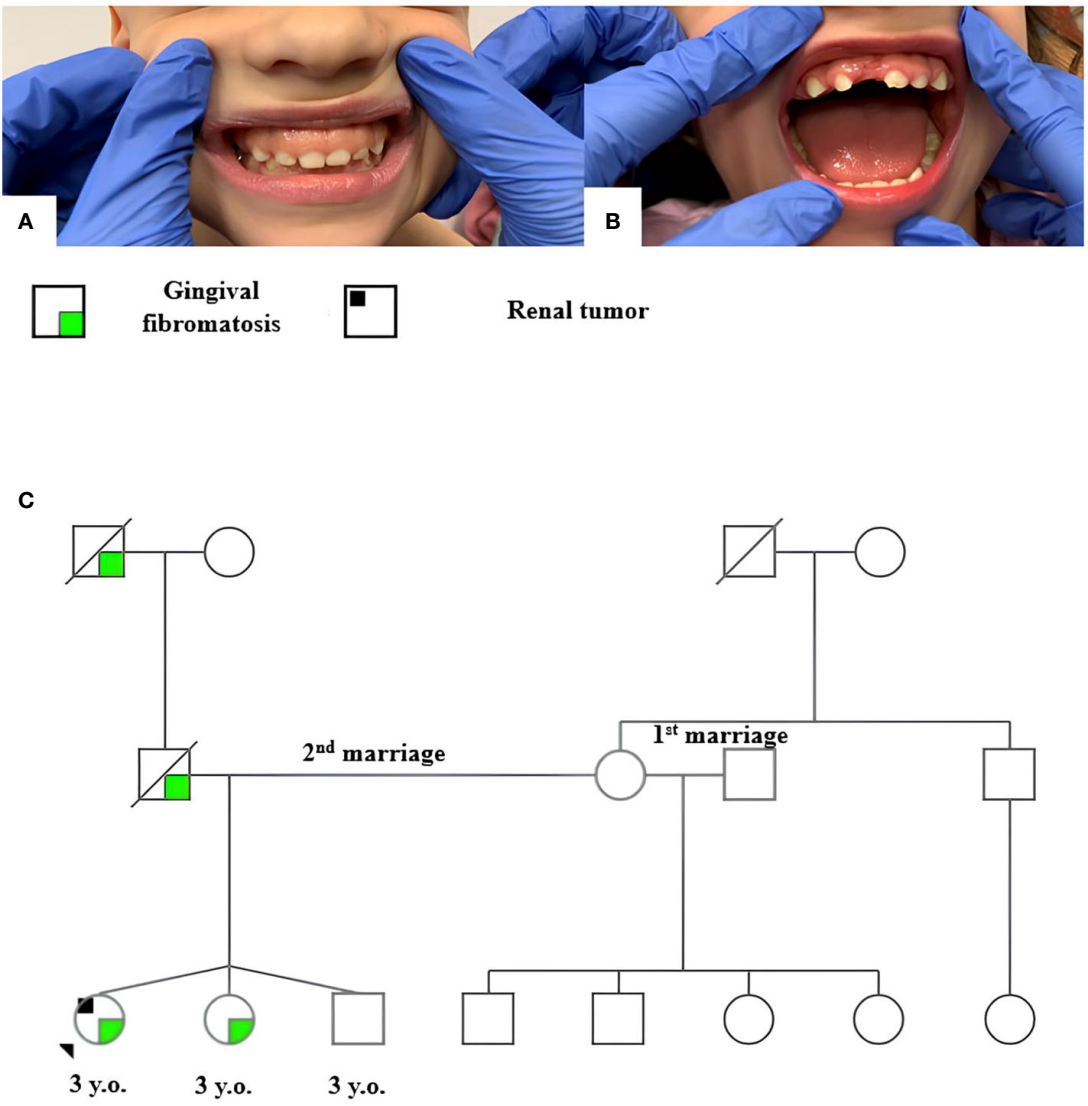
Gingival fibromatosis in patient 2 **(A)** and her sister **(B)**; family tree of patient 2 **(C)**.

The patient has no family history of cancer. Clinical examination revealed hyperplasia of the maxillary gums typical of GFM in the patient and her triplet sister (**Figures 2A, B**). The patient’s father, who died of a cause nonrelated to cancer, as well as their paternal grandfather, reportedly had similar changes in the upper jaw gums. The family tree is shown in **Figure 2C**.

The bilateral manifestation of tumors correlates with hereditary predisposition. NGS of peripheral blood DNA performed using the “tumor predisposition syndromes” panel (**Supplementary Table S1**) revealed the *REST* c.2668_2671del, p.E891Pfs*6 heterozygous mutation qualified as pathogenic. Subsequent genetic testing identified this constitutional variant in the patient’s sister.

Somatic NGS revealed only a missense variant in *DROSHA* (RefSeq NM_013235.5) c.3439G>A, p. E1147K, albeit for the left kidney tumor only. A search for copy number anomalies based on somatic NGS data revealed no extensive deletions in *REST* for tumors in both kidneys. Thus, the case presented a paternally inherited inactivating event in the *REST* coding sequence unaccompanied by somatic changes to the second, wild-type copy of the gene.

## Discussion

**Figure 3:**
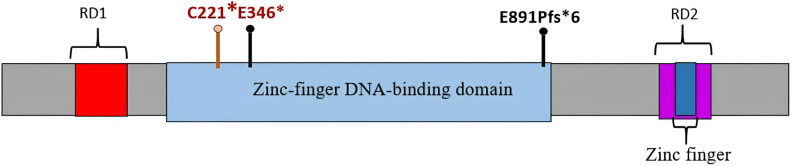
REST protein with zinc-finger DNA-binding domain and repressor domains RD1 and RD2. Germline mutations identified in patients 1 and 2 are marked with black pins; mutations (germline and somatic) identified in patient 1 are labeled in red.

The *REST* gene (OMIM *600571) encodes a Krüppel-type transcription factor with a zinc-finger DNA-binding domain and two repressor domains that interact with transcription apparatus and chromatin remodeling complexes (**Figure 3**). The protein inhibits the activation of neuron-specific genes in nonneural cells through chromatin remodeling (9, 10).

*REST* has been implicated as a putative tumor driver—either oncogene or suppressor, depending on the particular cancer type. Increased expression of *REST* in low-grade gliomas has been correlated to poor overall survival (11), which implicates it as a putative oncogene or disease progression marker in low-grade gliomas.

Germline-inactivating *REST* variants were for the first time associated with WT by Mahamdallie et al. (2015) describing a total of 11 such mutations in four nonrelated pedigrees with familial WT and nine patients with no family history of kidney tumors. Seven of 14 identified familial carriers had kidney tumors, which corresponds to a 50% life-long risk of renal cancer. In two cases, molecular examination revealed a second genetic event in *REST*, supporting its putative role as a suppressor in WT. Noteworthy, of the 11 identified genetic variants, 10 affected the DNA-binding domain of the REST protein (6).

One of our patients, patient 1, presented with a second, somatic, event in the *REST* coding sequence, apparently affecting the germline-intact copy of the gene. Such “double-hit” patterns feature *REST* as a tumor suppressor with the capacity of a single driver when double-inactivated. Patient 2 preserved the wild-type copy but presented with a missense *DROSHA* c.3439G>A, p. E1147K mutation previously characterized as truly somatic in WT (12). This second event was identified in residual tumor of the left kidney comprising epithelial foci but absent in blastema-only specimens.

According to Maciaszek et al. (2020), about 2% of WTs, regardless of histological type, harbor a germline pathogenic mutation in *REST*. The median age at diagnosis for such cases is 3 years (within the range of 0.5–6 years) (7). *REST* variants identified by us in two patients have not been described previously. Similarly, with the majority of WT-associated *REST* variants, these are loss-of-function mutations affecting the DNA-binding domain.

Familial GFM is a benign hyperplasia of fibrous tissues in the gingivae and periodontium. This genetically diverse condition has been associated with aberrations/mutations mapping to the *SOS1* gene, 5q13-q22 (OMIM #605544), 2p23.3-p.22.3 (OMIM #609955), and 11p15 (OMIM #6011010). The *REST* gene has been implicated as well: Bayram et al. (2017) describe three nonrelated families with GFM caused by three different heterozygous mutations in the last exon of *REST*, identified by whole-exome sequencing of peripheral blood DNA. Importantly, the article mentions no kidney tumors in any of the affected individuals (11 cases in total) (8).

Reciprocally, none of the articles on *REST* ^mut^ WT mention GFM as a comorbid condition. To the best of our knowledge, this report provides unique evidence on the WT-GFM comorbidity in *REST* ^mut^ carriers.

## Conclusion

The two cases illustrate Wilms tumor-gingival fibromatosis comorbidity in carriers of germline-inactivated *REST* alleles previously identified as a predisposition factor for both conditions. In our opinion, such comorbidities warrant immediate molecular testing for constitutional mutations in *REST*, with extremely important implications in terms of cancer risks. Moreover, clinical signs of gingival fibromatosis can justify ultrasound examination of the kidneys on a guideline basis in pediatric patients.

Despite the lack of comprehensive evidence on the pathogenetic role of *REST* in Wilms tumor, the loss-of-function nature of the variants implicates it as a putative suppressor. This assumption is supported by a second event affecting the germline-intact copy of *REST i*n a significant proportion of such tumors.

## Data availability statement

The original contributions presented in the study are included in the article/**Supplementary Material**. Further inquiries can be directed to the corresponding author.

## Ethics statement

The studies involving human participants were reviewed and approved by D. Rogachev Center Independent Ethics Committee. Written informed consent to participate in this study was provided by the participants’ legal guardian/next of kin. Written informed consent was obtained from the individual(s), and minor(s)’ legal guardian/next of kin, for the publication of any potentially identifiable images or data included in this article.

## Author contributions

AS, AD, MK, LY, and YM: conceptualized and drafted the initial manuscript. AS, AD, and MK: paper compilation and research. All authors contributed to the article and approved the submitted version.
